# 

**Published:** 1899-03

**Authors:** 


### THE BRISTOL MEDICO-CHIRURGICAL JOURNAL

**A Quarterly Journal of the Medical Sciences for the West of England and South Wales**

*PUBLISHED UNDER THE AUSPICES OF*

*THE BRISTOL MEDICO-CHIRURGICAL SOCIETY.*

LIBRARY SURGEON GENERAL'S OFFICE APR-24-1899

"Scire est nescire, nisi id me Scire alius sciret."

*Editor:* R. SHINGLETON SMITH, M.D., B.Sc.

WITH WHOM ARE ASSOCIATED

J. MICHELL CLARKE, M.A., M.D., W. H. HARSANT,

B. M. H. ROGERS, B.A., M.D., B.Ch., AND JAMES SWAIN, M.S., M.D.

*Assistant-Editor:* L. M. GRIFFITHS.

*Editorial Secretary:* JAMES TAYLOR.

BRISTOL: J. W. ARROWSMITH.

London: J. & A. Churchill, 7 Great Marlborough Street.

*Price One Shilling and Sixpence.*

